# A new ratio for better predicting future death/myocardial infarction than standard lipid measurements in women >50 years undergoing coronary angiography: the apolipoprotein A1 remnant ratio (Apo A1/ [VLDL_3_+IDL])

**DOI:** 10.1186/1476-511X-12-55

**Published:** 2013-04-26

**Authors:** Heidi T May, John R Nelson, Krishnaji R Kulkarni, Jeffrey L Anderson, Benjamin D Horne, Tami L Bair, Joseph B Muhlestein

**Affiliations:** 1 Intermountain Medical Center, Cardiovascular Department, 5121 S. Cottonwood Street, Murray, UT, 84157, USA; 2 UCSF, Fresno, CA, USA; 3 Atherotech, Birmingham, AL, USA; 4 University of Utah, Salt Lake City, UT, USA

**Keywords:** Lipid, Lipoprotein, Risk, Women, Outcomes, Apolipoprotein A1, Remnant lipoproteins

## Abstract

**Background:**

Women often lag behind men in their risk of cardiovascular events. However, with age and the onset of menopause, women’s cardiovascular risk eventually becomes similar to that of men. This change in risk may, in part, be attributable to a shift to a more atherogenic lipid profile. Our objective was to evaluate standard- and sub-lipid parameters and the apo A1 remnant ratio: (apo A1/[VLDL_3_-C+IDL-C]) for their associations with death/myocardial infarction among peri- and post-menopausal women.

**Methods:**

Women (N=711) >50 years of age undergoing coronary angiography were evaluated. Baseline clinical and angiographic characteristics, lipids, and sub-lipid levels (Vertical Auto Profile method) were collected. Cox regression analysis, adjusted by standard cardiovascular risk factors, was utilized to determine associations of lipid and sub-lipid tertiles(T) with death/myocardial infarction at 1 and 3 years.

**Results:**

Patients averaged 67.7±9.4 years and 53.6% had underlying severe (≥70% stenosis) coronary artery disease. The apo A1 remnant ratio was found to have stronger associations for 1 year (T1 vs. T3: HR=2.13, p=0.03, T2 vs. T3: HR=1.57, p=0.21) and 3 year (T1 vs. T3: HR=2.32, p=0.002, T2 vs. T3: HR=1.97, p=0.01) death/myocardial infarction than any individual lipid (LDL-C, HDL-C, triglycerides, non-HDL-C) or sub-lipid (apo A1, apo B, VLDL_3_-C+IDL-C) measure, or any other well-known ratio (triglyercies/HDL-C, apo B/A1, TChol/HDL-C, HDL-C/[VLDL_3_-C+IDL-C]).

**Conclusions:**

The apo A1 remnant ratio was a significant predictor of short and intermediate-term death/myocardial infarction risk among women >50 years of age. Furthermore, this ratio was found to have greater predictive ability than traditional lipid and sub-lipid parameters and represents a potential new risk marker.

## Background

Coronary heart disease (CHD) is the leading cause of death among men and women, although the risk in women temporally lags behind that of men (1). Nevertheless, the median survival for a woman age 55 to 64 with a first myocardial infarction (MI) is only 13.3 years compared to 17 years for a man [1]. Furthermore, for women aged 65 to 74 this median survival decreases to only 8.8 years [1]. Also, 64% of women who die suddenly of CHD have no previous symptoms compared to 50% for men. While most CHD risk factors are similar between men and women, they can often vary in strength and association and change throughout the lifetime [2,3]. This is especially evident for lipids, with low high-density lipoprotein cholesterol (HDL-C) cholesterol and hypertriglyceridemia being stronger predictors of CHD in women than in men [4-6]. Also, with age and the onset of menopause, women’s lipoprotein profile becomes more atherogenic [3], which is associated with a rapid increase in cardiovascular risk. However, how to best quantify cardiovascular risk in women incorporating lipid parameters is unclear.

While low-density lipoprotein cholesterol (LDL-C), an atherogenic lipoprotein, has been recognized as the target of lipid lowering therapy, it is not always indicative of atherogenicity. Other lipoprotein parameters (i.e., remnant lipoproteins) have been shown to be more atherogenic, particularly in women, including apolipoprotein (apo) B, intermediate-density lipoprotein cholesterol (IDL-C), and subfractions of very low-density lipoprotein cholesterol (VLDL-C) [7-11]. In addition, the triglyceride to HDL-C ratio (TG/HDL-C) has been shown to be an atherogenic marker [12] and a powerful independent predictor of all cause mortality and cardiovascular events among postmenopausal women [13] and men [14,15].

Gofman et al. first reported over 60 years ago that remnant lipoproteins were associated with the development of CHD [16]. Remnant lipoproteins have been shown to be elevated in patients with the metabolic syndrome [17], impaired glucose tolerance, and type 2 diabetes mellitus [18]. Remnant lipoproteins have also been reported to predict the extent of angiographically defined coronary artery disease (CAD) in post-menopausal women [19] and predict coronary events in patients with CAD [20,21].

It is well recognized that HDL-C has a strong inverse association with adverse CHD events among women. However, clinical events continue to occur even in patients who achieve “target” levels. The Framingham Heart Study reported that 43% of clinical events occurred in women with an HDL-C ≥50 mg/dL [6]. Also, apo A1, the major component of HDL, has been shown to predict short-term and long-term risk in patients with normal HDL-C [22]. This finding indicates that just measuring HDL-C may not be sufficient for ideal risk assessment. Therefore, alternative or complementary risk marker assessment, such as measuring HDL components (apo A1) and composition, for determining CHD risk may need to be utilized. The purpose of this study was to evaluate a novel lipid ratio: apo A1 remnant ratio (apo A1/[VLDL_3_-C+IDL-C]), which takes into account the importance of both apo A1 and remnant lipoproteins, with their association to death and myocardial infarction (MI) among peri and post-menopausal women. Furthermore, the utility of this new ratio was compared to traditional lipid parameters, apo A1, apo B, and the ratios total cholesterol to HDL-C (TChol/HDL-C), TG/HDL-C, apo B/A1, and HDL-C/(VLDL_3_-C+IDL-C).

## Results

### Patient characteristics

A total of 711 women met inclusion criteria and were evaluated for this study. Average lipid and lipoprotein levels among the entire cohort were (mg/dL): HDL-C: 48.3±15.6, LDL-C: 106.7±37.3, non-HDL-C: 136.5±41.3, apo B: 76.7±18.3, apo B/A1 ratio: 0.66±0.18, apo A1: 119.7±20.3, apo A1/(VLDL_3_-C+IDL-C): 6.6±3.6, TChol/HDL-C ratio: 4.3±2.8, VLDL_3_-C+IDL-C: 22.1±9.1, HDL-C/(VLDL_3_-C+IDL-C): 2.1±1.4, and LDL-C/HDL-C: 2.5±1.8. Triglycerides and the TG/HDL-C ratio were non-normally distributed and therefore, the median levels are reported: 133.0 mg/dL and 2.9, respectively. Patient characteristics for the cohort are shown in Table 1. A total of 41.3% of patients were discharged on a lipid lowering medication (37.1% were statins), 42.1% on aspirin, 27% on an ACEI, 17.4% on an ARB, and 33.9% on a beta-blocker.

**Table 1:** Baseline characteristics of study participants (N=711)

**Characteristic**	**Distribution**
Age (years)	67.7±9.4
Hypertension	64.6% (459)
Hyperlipidemia	49.9% (355)
Diabetes	21.4% (152)
Metabolic Syndrome	31.2% (222)
Family history of coronary artery disease	42.3% (301)
Smoking	12.7% (90)
Renal failure	1.0% (7)
Heart failure	0.8% (6)
Prior myocardial infarction	5.8% (41)
Prior cerebrovascular accident	4.6% (33)

### Long-term outcomes

**Figure 1:**
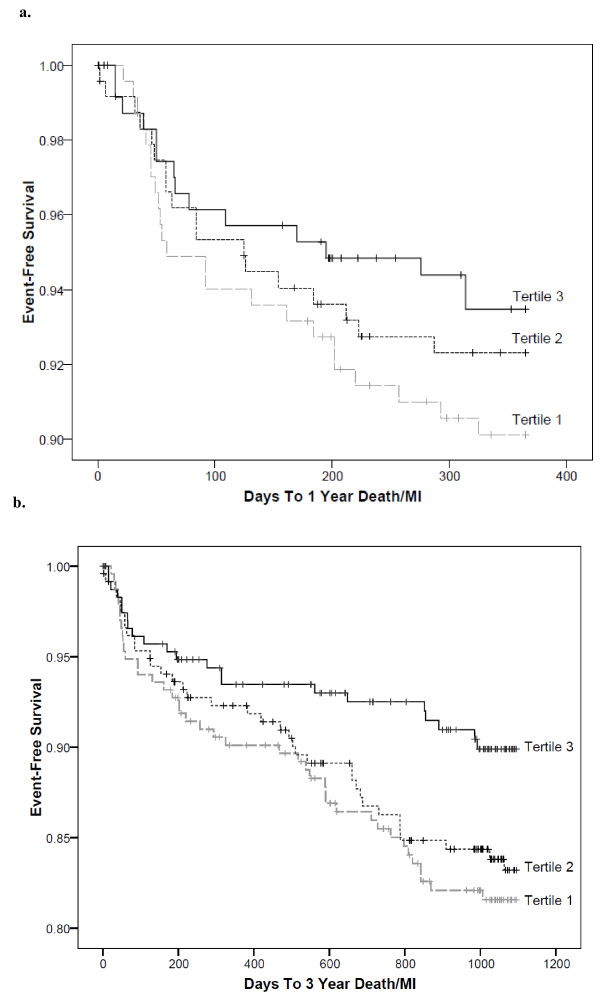
**Longitudinal associations of tertiles of the apolipoprotein A1 remnant ratio (apo A1/ [VLDL**_**3**_**-C+IDL-C]) at a) 1 year death/MI (log rank p-value=0.40) and b) 3 year death/MI (log rank p-value=0.04).**

Lipoprotein ratios and individual particles were the best predictors of short-term (1 year) and intermediate-term (3 year) death/MI risk (Table 2). After adjustment, there was a significant association between tertiles of the apo A1 remnant ratio and death/MI at both time points (Table 3). Tertile 1 for both apo A1 and HDL-C/(VLDL_3_-C+IDL-C) were significantly associated with increased risk at 3 years when compared to tertile 3. Though significance was not achieved at 1 year for death/MI for both apo A1 and HDL-C/(VLDL_3_-C+IDL-C), tertile 1 vs. tertile 3 had clinically meaningful hazard ratios (1.81 and 1.70, respectively). The remnants, VLDL_3_-C+IDL-C, were associated with 1 year death/MI for tertile 3 vs. tertile 1. Tertile 3 vs. tertile 1 for the apo B/A1 ratio was a significant predictor of death/MI at both 1 and 3 years. Longitudinal associations of tertiles of the apo A1 remnant ratio are displayed in Figure 1 for 1 year (a) and 3 year (b) death/MI. Though, the log rank p-value did not achieve significance at 1 year (most likely attributable to a lower event rate), there is a clear separation between the curves that show a distinct risk between the tertiles.

**Table 2:** Death/MI event rates at 1 and 3 years among lipid and lipoprotein tertiles

**Outcome**	**Tertile 1**	**Tertile 2**	**Tertile 3**	**p-trend**
**Apo A1/(VLDL**_**3**_**-C+IDL-C)**				
1 year Death/MI	9.7% (23)	7.6% (18)	6.3% (15)	0.17
3 year Death/MI	17.3% (41)	15.6% (37)	9.3% (22)	0.01
**Apo A1**				
1 year Death/MI	11.4% (27)	5.5% (13)	6.8% (16)	0.06
3 year Death/MI	21.1% (50)	9.3% (22)	11.8% (28)	0.004
**Apo B**				
1 year Death/MI	6.3% (15)	10.1% (24)	7.2% (17)	0.27
3 year Death/MI	13.9% (33)	16.0% (38)	12.2% (29)	0.49
**Apo B/A1**				
1 year Death/MI	7.2% (17)	5.1% (12)	11.4% (27)	0.03
3 year Death/MI	12.2% (29)	11.4% (27)	18.6% (44)	0.05
**HDL-C**				
1 year Death/MI	8.4% (20)	6.6% (15)	8.5% (21)	0.98
3 year Death/MI	18.6% (44)	10.2% (23)	13.3% (33)	0.10
**Triglycerides**				
1 year Death/MI	7.7% (18)	9.2% (22)	6.8% (16)	0.72
3 year Death/MI	12.3% (29)	15.4% (37)	14.4% (34)	0.52
**LDL-C**				
1 year Death/MI	8.3% (20)	8.2% (19)	7.1% (17)	0.63
3 year Death/MI	16.3% (39)	12.9% (30)	13.0% (31)	0.31
**non-HDL-C**				
1 year Death/MI	6.8% (16)	10.2% (24)	6.7% (16)	0.96
3 year Death/MI	14.0% (33)	16.6% (39)	11.7% (28)	0.46
**Triglycerides/HDL-C Ratio**				
1 year Death/MI	7.9% (23)	9.1% (21)	6.4% (12)	0.64
3 year Death/MI	11.6% (34)	17.2% (40)	13.9% (26)	0.37
**VLDL**_**3**_**-C+IDL-C**				
1 year Death/MI	6.8% (17)	7.4% (16)	9.3% (23)	0.29
3 year Death/MI	11.6% (29)	15.8% (34)	15.0% (37)	0.27
**Total Cholesterol/HDL-C Ratio**				
1 year Death/MI	8.9% (21)	7.6% (18)	7.2% (17)	0.78
3 year Death/MI	13.5% (32)	12.6% (30)	16.1% (38)	0.52
**HDL-C/(VLDL**_**3**_**-C+IDL-C)**				
1 year Death/MI	10.6% (25)	6.3% (15)	6.7% (16)	0.12
3 year Death/MI	19.1% (45)	13.0% (31)	10.1% (24)	0.005
**LDL-C/HDL-C**				
1 year Death/MI	9.7% (23)	5.5% (13)	8.4% (20)	0.61
3 year Death/MI	14.3% (34)	11.4% (27)	16.5% (39)	0.51

*Apo*: apolipoprotein; *HDL-C*: high density lipoprotein cholesterol; *IDL-C*: intermediate density lipoprotein cholesterol; *LDL-C*: low density lipoprotein cholesterol; *VLDL-C*: very low density lipoprotein_3_ cholesterol.

**Table 3:** Multivariable association of tertiles of lipoprotein and lipid parameters for 1 and 3 year Death/MI

**Outcome**	**1 year death/MI**	**3 year death/MI**
**Apo A1-C/(VLDL**_**3**_**-C+IDL-C)**		
Tertile 1 vs. Tertile 3	HR=2.14, p=0.03	HR=2.33, p=0.003
Tertile 2 vs. Tertile 3	HR=1.52, p=0.27	HR=1.95, p=0.02
**Apo A1**		
Tertile 1 vs. Tertile 3	HR=1.81, p=0.07	HR=1.80, p=0.02
Tertile 2 vs. Tertile 3	HR=0.77, p=0.49	HR=0.72, p=0.27
**Apo B**		
Tertile 2 vs. Tertile 1	HR=1.70, p=0.11	HR=1.18, p=0.49
Tertile 3 vs. Tertile 1	HR=1.34 p=0.42	HR=0.96, p=0.89
**Apo B/A1 Ratio**		
Tertile 2 vs. Tertile 1	HR=0.76, p=0.48	HR=0.94, p=0.82
Tertile 3 vs. Tertile 1	HR=1.97, p=0.03	HR=1.83, p=0.01
**HDL-C**		
Tertile 1 vs. Tertile 3	HR=0.96, p=0.89	HR=1.19, p=0.48
Tertile 2 vs. Tertile 3	HR=0.72, p=0.36	HR=0.62, p=0.10
**Triglycerides**		
Tertile 2 vs. Tertile 1	HR=1.56, p=0.20	HR=1.48, p=0.14
Tertile 3 vs. Tertile 1	HR=1.19, p=0.65	HR=1.26, p=0.40
**LDL-C**		
Tertile 2 vs. Tertile 1	HR=0.88, p=0.73	HR=0.74, p=0.26
Tertile 3 vs. Tertile 1	HR=1.35, p=0.38	HR=1.09, p=0.74
**Non-HDL-C**		
Tertile 2 vs. Tertile 1	HR=1.19, p=0.62	HR=1.11, p=0.69
Tertile 3 vs. Tertile 1	HR=1.30, p=0.47	HR=1.002, p=0.99
**Triglycerides/HDL-C Ratio**		
Tertile 2 vs. Tertile 1	HR=1.10, p=0.76	HR=1.53, p=0.08
Tertile 3 vs. Tertile 1	HR=0.90, p=0.77	HR=1.20, p=0.50
**VLDL**_**3**_**-C+IDL-C**		
Tertile 2 vs. Tertile 1	HR=1.31, p=0.45	HR=1.46, p=0.14
Tertile 3 vs. Tertile 1	HR=1.90, p=0.05	HR=1.55, p=0.09
**Total Cholesterol/HDL-C Ratio**		
Tertile 2 vs. Tertile 1	HR=0.89, p=0.72	HR=0.96, p=0.87
Tertile 3 vs. Tertile 1	HR=0.91, p=0.77	HR=1.27, p=0.33
**HDL-C/(VLDL**_**3**_**-C+IDL-C)**		
Tertile 1 vs. Tertile 3	HR=1.70, p=0.10	HR=2.07, p=0.004
Tertile 2 vs. Tertile 3	HR=0.97, p=0.92	HR=1.35, p=0.27
**LDL-C/HDL-C**		
Tertile 2 vs. Tertile 1	HR=0.60, p=0.15	HR=0.89, p=0.64
Tertile 3 vs. Tertile 1	HR=0.96, p=0.90	HR=1.15, p=0.57

*Apo*: apolipoprotein; *HDL-C*: high density lipoprotein cholesterol; *IDL-C*: intermediate density lipoprotein cholesterol; *LDL-C*: low density lipoprotein cholesterol; *VLDL-C*: very low density lipoprotein_3_ cholesterol.

### The Apo A1 remnant ratio: apo A1/ (VLDL_3_-C+IDL-C)

We evaluated whether we could better stratify risk prediction beyond tertiles. Therefore, using recursive partitioning, we determined that the cutoffs of ≤3.5 (category 1, n=98), >3.5-6.0 (category 2, n=310), and >6.0 (category 3, n=303) were the ideal categorizations for stratifying death/MI risk. The sensitivity and specificity at 3.5 (cutoff between categories 1 and 2) were 0.786 and 0.870 at 1 year and 0.80 and 0.872 at 3 year, respectively. For the cutoff of 6.0 (between categories 2 and 3), the sensitivity and specificity were 0.286 and 0.438 at 1 year and 0.270 and 0.452 at 3 years, respectively. Therefore, the categories were good discriminators between those at low and moderate-to-high risk. Baseline characteristics across categories of the apo A1 remnant ratio are shown in Table 4. Those in category 1 were more likely to have hypertension, hyperlipidemia, and diabetes; whereas, those in categories 2 and 3 had greater use of lipid lowering drugs, including statins. A better lipid profile was seen progressively across apo A1 remnant ratio categories.

**Table 4:** **Baseline characteristics among categories of the apo A1 remnant ratio (apo A1/ [VLDL**_**3**_**-C+IDL-C])**

**Characteristic**	**Category 1 (n=98)**	**Category 2 (n=310)**	**Category 3 (n=303)**	**p-value**
Age (year)	68.5±9.2	67.3±9.0	68.0±9.9	0.44
Hypertension	72.4%	70.6%	55.8%	<0.0001
Hyperlipidemia	60.2%	51.3%	45.2%	0.03
Diabetes	33.7%	20.6%	18.2%	0.005
Metabolic Syndrome	53.1%	32.6%	22.8%	<0.0001
Family History of CAD	45.9%	42.9%	40.6%	0.63
Smoking	10.2%	14.5%	11.6%	0.40
Prior CAD	44.0%	36.1%	38.2%	0.43
Prior MI	5.1%	6.5%	5.3%	0.79
Presentation				0.53
Stable Angina	59.2%	65.2%	60.1%	
Unstable Angina	30.6%	23.5%	29.0%	
MI	10.2%	11.3%	10.9%	
CAD	44.9%	41.9%	44.2%	0.80
Any Lipid Lower	27.4%	45.2%	42.0%	0.03
Statin	23.3%	42.1%	36.6%	0.02
Aspirin	42.5%	40.7%	43.4%	0.85
ACE Inhibitor	30.1%	31.2%	21.4%	0.06
ARB	16.4%	19.7%	15.1%	0.44
Beta-blocker	39.7%	32.3%	33.5%	0.50
LDL-C (mg/dL)	119.9±48.9	106.4±35.1	102.7±34.2	<0.0001
HDL-C (mg/dL)	41.7±14.9	46.1±13.7	52.7±16.6	<0.0001
Triglycerides (mg/dL)*	176.0	145.0	116.0	<0.0001
Apo A1 (mg/dL)	106.2±14.6	115.0±17.0	128.9±20.9	<0.0001
Apo B (mg/dL)	93.4±21.2	78.5±15.6	69.4±15.6	<0.0001
Apo B/Apo A1 Ratio	0.89±0.19	0.69±0.13	0.55±0.14	<0.0001
non-HDL-C (mg/dL)	158.0±55.1	137.7±37.3	128.2±37.2	<0.0001
Triglycerides/HDL-C Ratio*	4.7	3.2	2.3	<0.0001
VLDL_3_-C+IDL-C (mg/dL)	37.1±7.6	24.6±5.1	14.6±3.8	<0.0001
Total Cholesterol/HDL-C Ratio	5.1±1.6	4.4±2.9	3.9±2.9	<0.0001

*Median reported due to non-normal distribution.

*Apo*: apolipoprotein; *ARB*: angiotensin receptor blocker; *CAD*: coronary artery disease; *HDL-C*: high density lipoprotein cholesterol; *IDL-C*: intermediate density lipoprotein cholesterol *LDL-C*: low density lipoprotein cholesterol; mg/dL: milligrams/deciliter; *MI*: myocardial infarction; *VLDL-C*: very low density lipoprotein_3_ cholesterol.

Categories were determined by recursive partitioning and were stratified into the following: category 1: ≤3.5 mg/dL; category 2: 3.5-6.0 mg/dL; category 3: >6.0 mg/dL.

**Figure 2:**
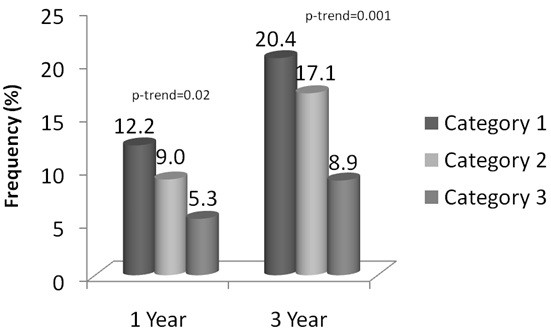
**Death/MI event rates at 1 and 3 years among categories of the apolipoprotein (apo) A1 remnant ratio (apo A1/ [VLDL**_**3**_**-C+IDL-C]).** * Category 1 (≤3.5), Category 2 (>3.5-6.0), and Category 3 (>6.0).

**Figure 3:**
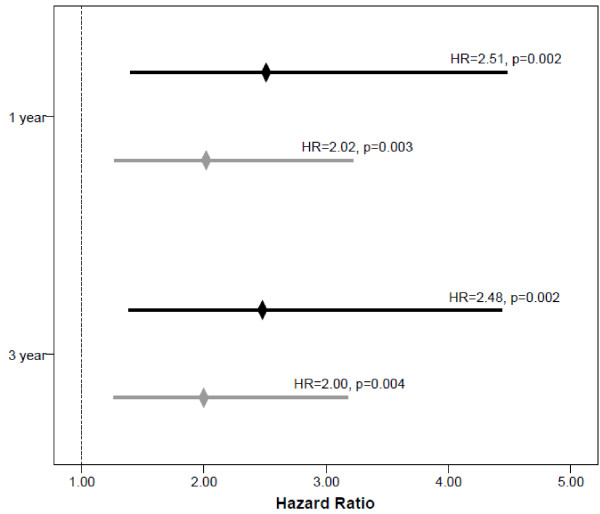
**Adjusted hazard ratios (HR) of categories of the apo A1 remnant ratio (apo A1/ [VLDL**_**3**_**-C+IDL-C]) at 1 year and 3 year death/MI.**

**Figure 4:**
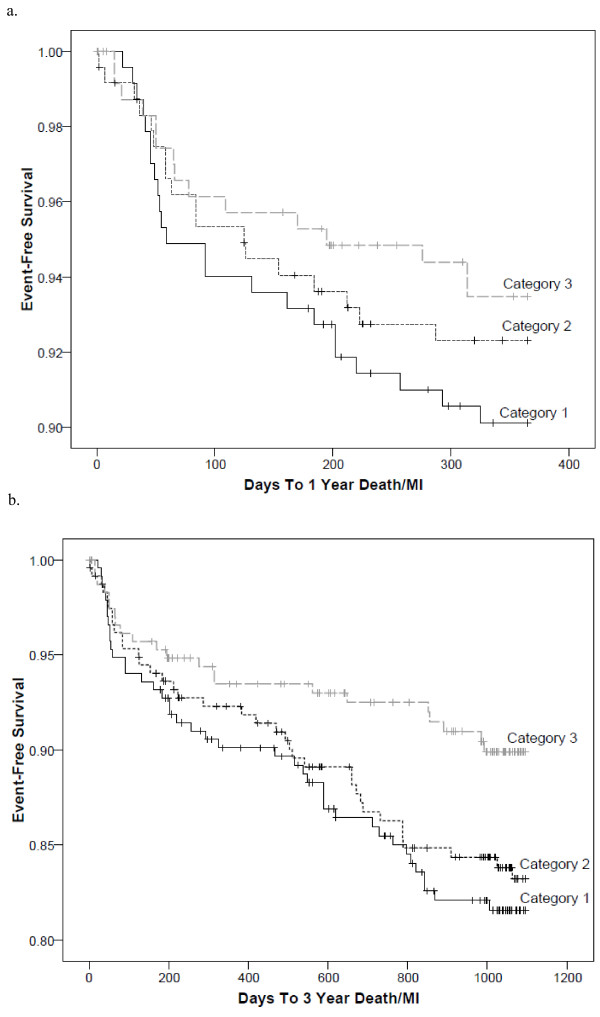
**Longitudinal associations of categories of the apo A1 remnant ratio (apo A1/ [VLDL**_**3**_**-C+IDL-C]) at a) 1 year death/MI (log rank p-value=0.40) and b) 3 year death/MI (log rank p-value=0.04).** * Category 1 (≤3.5), Category 2 (>3.5-6.0), and Category 3 (>6.0).

Figure 2 displays the frequency of events among the categories of the apo A1 remnant ratio, with a significant trend of increasing risk with decreasing levels. After adjustment, all category comparisons at both timepoints significantly predicted death/MI risk (Figure 3). When comparing tertile 1 to tertile 3 and tertile 2 to tertile 3, there was an approximate 2.5-fold and a 2-fold increase in risk for death/MI at both timepoints, respectively. These associations remained with little attenuation after adjustment by LDL-C, HDL-C, and non-HDL-C. Log-transformed triglycerides (r=-702), apo A1 (r=0.510), apo B/A1 (r=-0.58), and VLDL_3_-C+IDL-C (r=-0.76) were significantly correlated with the apo A1 remnant ratio and therefore could not be evaluated in the multivariable model. Longitudinal associations for 1 year and 3 year death/MI are shown in Figure 4. As with the tertiles, the log rank p-value for the apo A1 remnant ratio categories did not achieve significance at 1 year, however the curves do show a distinct difference in risk between the categories. The predictive ability of the apo A1 remnant ratio was similar among women when stratified by age (50–64 vs. ≥65 years) and CAD status.

## Discussion

This study presents for the first time in women greater than 50 years of age that the novel apo A1 remnant ratio (apo A1/[VLDL_3_-C+IDL-C]) is a strong predictor of death/MI at 1 and 3 years. This is seen among both tertiles, as well as from categories derived from recursive partitioning. Furthermore, this ratio had greater predictive ability than traditional lipid parameters, apo A1, apo B, and ratios such as TChol/HDL-C, TG/HDL-C, apo B/A1, and HDL-C/(VLDL_3_-C+IDL-C). The power of this ratio is related to both the numerator (apo A1) and the remnant denominator (VLDL_3_-C+IDL-C).

There are potentially important clinical ramifications related to this new ratio. As women age, LDL-C and HDL-C levels decline, indicating a change in HDL composition [3]. It has been shown that the specific composition of HDL alters its capacity to participate in reverse cholesterol transport and modulate inflammation. Therefore, measurements of traditional lipid parameters may not be sufficient in risk prediction. This study reinforces the growing body of evidence that among post- menopausal women, LDL-C, which is the current target of lipid therapy by the National Cholesterol Education Panel Adult Treatment Panel (NCEP ATP) III report [23], is not the most predictive lipid parameter for CHD risk [5,24]. In addition, remnant lipoproteins are known to be elevated in postmenopausal women [8] and emphasis on remnant lipoproteins has been highlighted in the NCEP ATP III guidelines, which recommends the calculation of non-HDL-C as its measure [24]. Although non-HDL-C provides a measure of remnant lipoproteins, it also is a measure of triglyceride-rich particles, which are less atherogenic. Another advantage of the apo A1 remnant ratio is that fasting is not required for sub-fraction measurements and therefore it could be precisely calculated. Therefore, the superiority of our ratio is not surprising since it utilizes the advantages of direct measurements of apo A1 and the remnant lipoproteins over traditional lipid parameters.

Apo A1, which is the major protein component of HDL, is largely responsible for reverse cholesterol transport through the macrophage ATP-binding cassette transporter ABCA1. Oxidative mechanisms can damage apo A1, which decreases its ability to effectively promote cholesterol efflux. Such modifications can convert HDL from an anti-inflammatory to a pro-inflammatory particle. Measuring HDL-C is indicative of the size of the HDL pool, but not its composition. Therefore, an advantage of utilizing apo A1 over HDL-C is that it reflects the functioning-state of the molecule. Though epidemiological studies have consistently shown HDL-C to be inversely correlated with atherosclerotic events [25,26], particularly among women [4], many events continue to occur despite reaching target levels [6]. Thus, emphasizing the need to determine additional measurements that can identify risk among “low-risk” populations. Further, it appears that HDL-C has a U-shaped distribution with a threshold for the upper limit of normal not being consistently defined for identifying risk, whereas apo A1 has a constant inverse relationship with CHD events [26]. Also, dysfunctional HDL is not always reflected in the HDL-C pool [27]. Unlike apo A1, very high values of HDL-C and HDL particle size have been shown to be associated with increased major adverse cardiovascular event risk [28].

We found low levels of apo A1 to be associated with an 80% increase in 1 and 3 year death/MI risk, whereas levels of HDL-C were found to not be predictive. The apo A1 remnant ratio utilizes the predictive value of apo A1, which was found to be highly predictive of death/MI by tertiles and by categories. We evaluated whether HDL-C would elicit similar results as apo A1 when divided by the remnant lipoproteins since a previous investigation found the inverse of this ratio, (VLDL_3_–C+IDL-C)/HDL-C, to be predictive of CHD [29]. We found the HDL-C/(VLDL_3_-C+ IDL-C) ratio, though predictive, to not achieve the same degree of predictive significance as the apo A1 remnant ratio. These results reinforce the importance of measuring apo A1, potentially above that of HDL-C, for determining death/MI risk among women >50 years.

Remnant lipoproteins are generated by lipolysis of triglyceride-rich lipoproteins and have been shown to play a greater role in CHD development than their precursors [11]. The Framingham Heart Study found remnant lipoproteins to be significantly higher in women with CHD when compared to women without CHD [8]. Among postmenopausal women, Lamon-Fava et al. reported that atherosclerosis severity is linked to a dysregulation of triglyceride/HDL metabolism [19]. They hypothesized that increases in remnant lipoproteins may be associated with increased lipid deposition in the arterial wall, plaque formation, and the generation of HDL-C subpopulations that are less efficient in the reverse cholesterol removal from the arterial wall [19]. Remnant lipoproteins, independent of cardiovascular risk factors, have also been shown to be associated with peripheral vascular endothelial dysfunction as measured by Doppler flow analysis [30], with this being more pronounced in the postprandial state [31]. Furthermore, remnant lipoproteins directly promote the initial stages of atherosclerosis through regulating the expression of ICAM-1 and VCAM-1, which are required for attachment of monocytes to the endothelial wall [32]. In addition, they have been shown to promote a pro-coagulant state and induce tissue factor in cultured endothelial cells, with large and small VLDLs supporting factor Xa and factor Xa/Va-mediated factor VII activation [32,33]. Finally, VLDL particles generate thrombin at rates near that of activated platelets [34]. These pro-inflammatory, pro-atherogenic, and pro-thrombotic effects of remnant lipoproteins, which are independent from traditional risk factors including LDL-C, further explain the increased event rate seen in patients with CHD and increased remnant lipoprotein levels [35]. Finally, it is possible that remnant lipoproteins may be the driving factor of the increased CHD risk seen in middle aged and older woman with elevated triglycerides [36-41]. Within our study, remnant elevations were found to be associated with 1 year death/MI (HR=1.90, p=0.05), with a trend toward significance for 3 year death/MI risk (HR=1.55, p=0.09). Triglycerides were not associated with death/MI risk.

Many lipid and sub-lipid ratios have been reported to predict CHD risk. In the Women’s Ischemia Syndrome Evaluation (WISE) study, the TG/HDL-C ratio was found to predict cardiovascular events and mortality among women without prior MI or coronary revascularization, but non-HDL-C, the TChol/HDL-C ratio, and total cholesterol were not [13]. However, the TG/HDL-C was no longer predictive of cardiovascular events when adjusted by coronary artery disease severity. Within our study the apo A1 remnant ratio maintained its significance after adjustment by CHD severity. The TG/HDL-C ratio has also been shown to be predictive of cardiovascular events among other populations [14,15]. However, we found the TG/HDL-C ratio to be not predictive of 1 or 3 year death/MI among women >50 years.

The apo B/A1 ratio was shown in INTERHEART, a large, international case–control study, to be one of the most predictive parameters for identifying CHD risk [9]. It was also found to show a graded relationship with MI risk, with no evidence of a threshold. Of all of the risk factors evaluated, the apo B/A1 ratio contributed the greatest to the population attributable risk, even being superior to smoking. When stratified by sex and age, similar associations persisted across all sub-groups. AMORIS showed that apo B, apo A1, and the apo B/A1 ratio are important risk factors for fatal MI, even after adjustment by age, total cholesterol, and triglycerides, with the apo B/A1 ratio being the greatest predictor of risk [7]. These results were consistent among both men and women, young (<70 years) and old (≥70 years). The apo B/A1 ratio was predictive of 1 year and 3 year death/MI when the highest tertile was compared to the lowest tertile within our study, though its predictive power was not as strong as the apo A1 remnant ratio.

In a recent meta-analysis that evaluated 233,455 subjects with 22,950 events, apo B was found to be superior to non-HDL-C, which in turn was superior to LDL-C in predicting cardiovascular risk (RR=1.43, 1.34, 1.25, respectively) [42]. Furthermore, this study found that using an apo B strategy to treat those individuals at >70th percentile in the US adult population, 500,000 more events would have been prevented than if a non-HDL-C stategy had not been used. It has also been reported that apo B is a more accurate marker of cardiovascular risk than LDL-C, and in its absence, non-HDL-C should be used since it indirectly estimates apo B [43]. Our study showed that the apo A1 remnant ratio was more predictive than apo B or non-HDL-C. One explanation for this is that remnant lipoproteins are more atherogenic than other larger apo B containing triglyceride rich lipoproteins. Remnants are smaller particles and can diffuse into the arterial wall [44] and are more likely to be bound by heparin sulfate proteoglycans within the arterial media [45]; therefore, able to promote more events.

Results of this study seem to suggest that lipids, or some of their components, might not be the best indicator of increased risk and those with a greater concentration of denser, more atherogenic particles are at higher risk than in those with abnormal lipid concentrations. Therefore, the apo A1 remnant ratio may be a parameter that may better stratify risk of women >50 years so that those found in the higher risk categories can be more aggressively treated. Interventions may include earlier introduction or more aggressive lipid-lowering therapy. In this study, there was a significant difference between the use of lipid lowering medications among the categories at baseline, though lipid lowering use was adjusted for in the multivariable model, use of these medications could have affected which stratification, and thus risk of the patient. Future studies should evaluate longitudinal use of lipid lowering therapy on apo A1 remnant ratio levels. The apo A1 remnant ratio is indicative of the number of anti-atherogenic particles over the number of atherogenic particles. Thus, particle numbers and their ratios might be more important factors than the amount of lipids carried per particle.

### Study limitations

This study shares the limitations of all nonrandomized, observational studies, including the possibility of selection bias and unadjusted confounding. The population studied was high-risk (i.e., undergoing angiography), predominantly white, and women >50 years of age; thus these results may not apply to lower-risk populations, men, women ≤50 years, and other ethnic groups. The menopausal status in all women was not known; however approximately 80% of women over the age of 50 are either peri- or post-menopausal [46]. Therefore, the lipid changes associated with menopause might not be seen among all of the women and be non-randomly distributed across tertiles and categories. Further, we did not know the status of hormone replacement therapy, which positively affects lipid and lipoprotein profiles [47]. It is possible that there is a significant difference in the use of these medications across the lipid and lipoprotein tertiles and categories, which could have possible effects on the associations. We did not know the insulin resistance status of our patients, as insulin levels were not available to provide a calculation of HOMA-IR. However, 31.2% of our patients had metabolic syndrome and 21.4% had diabetes mellitus, with the apo A1 remnant ratio remaining statistically significant despite adjustment. The results of this study were confined to “hard” cardiovascular end points (death/MI); thus, we cannot give conclusions regarding “softer” cardiovascular endpoints such as angina, hospitalization, and revascularization.

## Conclusions

The new parameter, the apo A1 remnant ratio, was found to be a significant predictor of short-term and intermediate-term death/MI risk among women >50 years of age and was more strongly associated with death/MI risk than traditional lipid parameters, apo A1, apo B, and well-recognized lipid ratios. Further, this new ratio brings attention to the role of remnant lipoproteins in women greater than 50 years of age and represents a potential new measurement of risk. The apo A1 remnant ratio should be evaluated as a potential new treatment target and should be tested in a larger cohort and among other populations.

## Methods

### Study patients

Study patients were drawn from the Intermountain Heart Collaborative Study (IHCS) registry from March, 1999 to January, 2007 [48,49]. The IHCS is a cardiac catheterization registry that includes consenting patients undergoing catheterization at a major Intermountain Healthcare hospital (LDS Hospital: Salt Lake City, UT; Intermountain Medical Center: Murray, UT; and McKay Dee Hospital: Ogden, UT). At the time of angiography, patients that provided informed consent had a blood sample taken and stored cryogenically for future testing. Patients (N=711) were included in this study if they were female, >50 years of age, gave written informed consent, and had both lipid panel results available from hospitalization and lipoprotein subfraction measurements.

The Intermountain Healthcare Urban Central Institutional Review Board approved this study.

### Lipid and lipoprotein particle quantification and stratification

Lipid levels were obtained during angiography hospitalization at the discretion of the attending cardiologist. Total cholesterol and triglycerides were quantified using dry-slide measurement on the VITROS 950 Analyzer (Ortho Clinical Diagnostics; Rochester, NY). HDL-C was measured with the same instrument following treatment with VITROS HDL-Cholesterol Magnetic Reagent. LDL-C and VLDL-C were calculated from the total cholesterol and triglyceride measurements.

At the time of angiography, blood samples were collected in EDTA and refrigerated at 4°C. Within 24 hours, samples were centrifuged and plasma and DNA were separated and stored cryogenically. Cholesterol concentrations of lipoprotein classes and their subclasses were measured on samples obtained at angiography using single vertical spin density gradient ultra-centrifugation based on the Vertical Auto Profile (Atherotech, Birmingham, AL) [50]. Apo A1 was estimated by the VAP method using the procedure described previously [51].

Lipids and lipoproteins were evaluated as tertiles. Tertile stratifications for the different parameters are displayed in Table 5. For the apo A1 remnant ratio, apo A1, HDL-C, and HDL-C/(VLDL_3_-C+IDL-C) higher levels are desirable. However, lower levels are desired for triglycerides, LDL-C, non-HDL-C, apo B, apo B/A1 ratio, TG/HDL-C ratio, VLDL_3_-C+IDL-C, TChol/HDL-C ratio, and the LDL-C/HDL-C ratio.

**Table 5:** Lipid and lipoprotein tertile stratifications

	**Tertile 1**	**Tertile 2**	**Tertile 3**
Apo A1/(VLDL_3_-C+IDL-C)	<4.7	4.7-6.8	>6.8
Apo A1	<109.7	109.7-127.2	>127.2
Apo B	<67.3	67.3-81.9	>81.9
Apo B/A1 Ratio	<0.57	0.57-0.71	>0.71
HDL-C	<40	40-52	>52
Triglycerides	<113	113-160	>160
LDL-C	<88	88-117	>117
Non-HDL-C	<119	119-146	>146
Triglycerides/HDL-C Ratio	<2.3	2.3-3.7	>3.7
VLDL_3_-C+IDL-C	<17	17-24	>24
Total Cholesterol/HDL-C Ratio	<3.4	3.4-4.4	>4.4
HDL-C/(VLDL_3_-C+IDL-C)	<1.4	1.4-2.1	>2.1
LDL-C/HDL-C	<1.8	1.8-2.7	>2.7

*Apo*: apolipoprotein; *HDL-C*: high density lipoprotein cholesterol; *IDL-C*: intermediate density lipoprotein cholesterol; *LDL-C*: low density lipoprotein cholesterol; *VLDL-C*: very low density lipoprotein_3_ cholesterol.

### Other risk factor, demographic, and clinical assessments

In addition to age and gender, patient information collected included diabetes status (diabetes mellitus: fasting blood glucose > 125 mg/dL, clinical diagnosis of diabetes mellitus, or anti-diabetic medication use; insulin resistance: fasting glucose between 110–125 mg/dL; and normal: fasting glucose <110 mg/dL), hypertension (systolic blood pressure ≥ 140 mmHg, diastolic ≥ 90 mmHg, or anti-hypertensive use), renal failure (clinical diagnosis or GFR <15 ml/min), hyperlipidemia (total cholesterol ≥ 200 mg/dL, LDL ≥ 130 mg/dL, or cholesterol-lowering medication use), and congestive heart failure (clinical diagnosis or physician-reported). The metabolic syndrome was defined using ATP III criteria [52], with BMI >27 kg/m^2^ used as a surrogate for waist circumference. Family history was patient-reported if a first-order relative had suffered cardiovascular death, MI, or coronary revascularization before age 65 years. Smoking included active smokers or those with a >10 pack-year history. Clinical presentation included stable angina (stable exertional symptoms only), unstable angina (progressive symptoms or symptoms at rest), or acute MI (creatine kinase-MB >6 mg/dL and creatine kinase-MB index >3% or a troponin I level >0.4 ng/mL). Treatment type was defined as treatment with medication only, percutaneous coronary intervention, or coronary artery by-pass surgery. Admission and discharge medications (i.e., statin, other lipid lowering medications, ace-inhibitors [ACEI], aspirin, angiotensin receptor blocker [ARB], beta-blocker, clopidogrel, diuretic) were also available.

Significant CAD was defined as the presence of one or more ≥ 70% obstructive lesions by coronary artery angiography. Assessment of CAD was made by review of angiograms by the patient’s cardiologist, and results were entered into the computer database in a format modified after the coronary artery surgery study protocol [49]. On the basis of this angiographic evaluation, the patients were determined to have single-, double-, or triple-vessel disease as defined by the presence of a 70% stenosis in each major vessel counted, with the left main counting as two vessels. Assessment of CAD was performed blinded to results of blood testing.

### Patient follow-Up and event assessment

Average length of follow-up was 3.9±2.1 years. Events included the composite outcome of death and non-fatal MI at 1 and 3 years. MI was defined as a hospitalization where a patient had a troponin I level ≥0.4 ng/mL or a discharge diagnosis of an MI (ICD-9 code 410). Deaths were determined by telephone survey, hospital records, and Utah State Health Department records (death certificates) and were verified through Social Security death records. Patients not listed as deceased in any registry were considered to be alive.

### Statistical analysis

The chi-square test, Student’s *t* test, and the analysis variance (ANOVA) were used to examine lipid and lipoprotein tertiles to baseline characteristics. To confirm the associations of the composite outcome of death/MI determined by univariable analysis, multivariable Cox hazard regression (SPSS, version 15.0; Chicago, IL) was performed to determine hazard ratios (HRs). Kaplan-Meier survival estimates and the log rank test were used to determine initial associations with 1 and 3 year death/MI. Available baseline risk factors used in the modeling included age, gender, hypertension, hyperlipidemia, diabetes status, smoking, family history of CAD, renal failure, prior MI, prior cerebrovascular accident, congestive heart failure, presentation (stable angina, unstable angina, or acute MI), number of vessels with stenosis ≥70%, and discharge medications. Final models entered significant (p<0.05) and confounding (10% change in beta-coefficient) covariables. The proportional hazards assumption was met for all models. Two-tailed p-values are presented with 0.05 designated as nominally significant.

## Abbreviations

Apo: Apolipoprotein; MI: Myocardial infarction; T: Tertile; CHD: Coronary heart disease; HDL-C: High-density lipoprotein cholesterol; LDL-C: Low-density lipoprotein cholesterol; IDL-C: Intermediate-density lipoprotein cholesterol; VLDL-C: Very low-density lipoprotein cholesterol; TG: Triglyceride; CAD: Coronary artery disease; TChol: Total cholesterol; IHCS: Intermountain Heart Collaborative Study; ACEI: Ace-inhibitors; ARB: Angiotensin receptor blocker; ANOVA: Analysis variance; HR: Hazard ratio; NCEP ATP: National Cholesterol Education Panel Adult Treatment Panel

## Competing interests

HTM: none. JRN: consultant for Atherotech. KRK: employee of Atherotech. JLA: none. BDH: none. TLB: none. JBM: none.

## Authors’ contributions

HTM participated in the study design, performed the statistical analysis, and the writing of the manuscript. JRN participated in the study design and writing of the manuscript. KRK participated in the study design and critical revision of the manuscript. JLA participated in the critical revision of the manuscript. BDH participated in the critical revision of the manuscript. TLB participated in data acquisition and the critical revision of the manuscript. JBM participated in the study design and critical revision of the manuscript. All authors read and approved the final manuscript.
